# Adaptation processes in the auditory system of a beluga whale *Delphinapterus leucas*

**DOI:** 10.1371/journal.pone.0201121

**Published:** 2018-07-26

**Authors:** Vladimir V. Popov, Dmitry I. Nechaev, Alexander Ya. Supin, Evgeniya V. Sysueva

**Affiliations:** Laboratory of the Sensory Systems of Vertebrates, Institute of Ecology and Evolution, Russian Academy of Sciences, Moscow, Russia; Universidad de Salamanca, SPAIN

## Abstract

The effects of prolonged sound stimuli (tone pip trains) on evoked potentials (the rate following response, RFR) were investigated in a beluga whale. The stimuli (rhythmic tone pips) were of 64 kHz frequency at levels from 80 to 140 dB re 1 μPa. During stimulation, every 1000 ms stimulus level either was kept constant (the steady-state stimulation) or changed up/down by 20 or 40 dB. With such stimulus presentation manner, RFR amplitude varied as follows. (i) After a stimulus level increase, the response amplitude increased quickly and then decayed slowly. The more the level increased, the higher the response amplitude increased. (ii) After a stimulus level decrease, the response amplitude was suppressed and then recovered slowly. The more the level decreased, the stronger was the response suppression. (iii) At the end of the 1000 ms window, the response amplitude approached, but did not reach, the amplitude characteristic of the steady-state stimulation. As a result, both after a sound level increase and decrease, the responses were almost stabilized during an analysis time as short as 1 s. This stabilization is attributed to an adaptation process. RFR decay after initial increase could be approximated by an exponent with a time constant of 59.4 ±1.8 (standard error) ms; RFR recovery after initial decrease could be approximated by an exponent with a time constant of 139.2 ±9.9 ms.

## Introduction

Adaptation refers to the temporary modifications in the response properties of neurons of the auditory system induced by a recent or current stimulus, particularly during or after a prolonged sound. The classical effects of adaptation have been described as a reduction of neuronal activity and response to test stimuli during the presentation of an adaptive sound. In laboratory animals, it has been observed that the responses of auditory nerve fibers [1–7], as well as the cochlear action potential [8], decrease during the presentation of long auditory stimuli.

Auditory adaptation affords an adjustment of the dynamic range of the auditory system to the mean sound pressure level (SPL) of the acoustic environment. This adjustment enables the maintenance of high differential sensitivity within a wide range of sound levels (more than 100 dB), whereas the dynamic range of auditory neurons, as a rule, is as narrow as 20–30 dB [9, 10, 11].

The described adaptation processes can be approximated by a combination of several exponents with time constants of approximately 5 ms, 20–40 ms, and seconds or tens of seconds [2, 3, 12]. These estimates are important for understanding the physiological nature of the adaptation processes. However, these estimates of the adaptation time course characterize a limited spectrum of mammal species that are typically used as laboratory animals. Meanwhile, mammals include a wide variety of species featuring various specializations of the auditory system. In particular, the auditory system of odontocetes (toothed whales, dolphins, and porpoises) is of special interest because it features not only a wide (more than 100 kHz) frequency range but also a considerably higher temporal resolution than many other mammals [13, 14, 15]. An investigation of the adaptation processes in an auditory system with such unique features might expand the knowledge of the mechanisms of auditory adaptation.

A first attempt has been made to investigate auditory adaptation in the auditory system of an odontocetes representative, the beluga whale *Delphinapterus leucas* [16]. We showed that prolonged exposure to a sound signal results in a progressive reduction of the response magnitude. These data demonstrated the effects of adaptation within a wide range of signal durations: the reduction of the responses appeared several milliseconds after the onset of stimulation and continued to grow for longer than 1 h. This process could be approximated by at least two exponents with different time constants: 30–80 ms and 3.1–17.6 s. Our previous study presented the effect of adaptation for a particular case in which adaptation was revealed by responses to the same signal that produced the adaptation. However, the adaptive change of hearing sensitivity produced by a given long-lasting signal should also affect the perception of other signals, both of a lower and higher level than the adapting signal. The features of this effect have not been investigated in odontocetes to date, which was the goal of the present study.

To achieve this goal, we recorded auditory evoked potentials (AEP) to auditory stimuli in a beluga whale. Specifically, rhythmic trains of tone pips were used as stimuli, and AEPs to these trains, which is known as the rate-following response (RFR), were recorded and analyzed.

## Methods

### Subject and experimental design

The subject was an adult female beluga whale, *D*. *leucas*, kept at the Utrish Marine Station of the Russian Academy of Sciences on the Black Sea coast. The animal was housed in a round sea water tank that was 6 m in diameter and 1.5 m in depth. The care and use of the animal were in compliance with the guidelines of the Russian Ministry of Education and Science for the use of animals in biomedical research. The study was approved by the Ethical committee of the Institute of Ecology and Evolution.

During experimentation, the water level in the pool was lowered to 60 cm. The animal was supported by a stretcher such that the dorsal part of the body and the blowhole were above the water surface. The stretcher was made of fish net and was transparent to sound. The animal was not anesthetized. The transducer that served to play sounds was immersed in the water at a depth of 30 cm, 1 m in front of the animal’s head.

### Test stimuli

**Fig 1:**
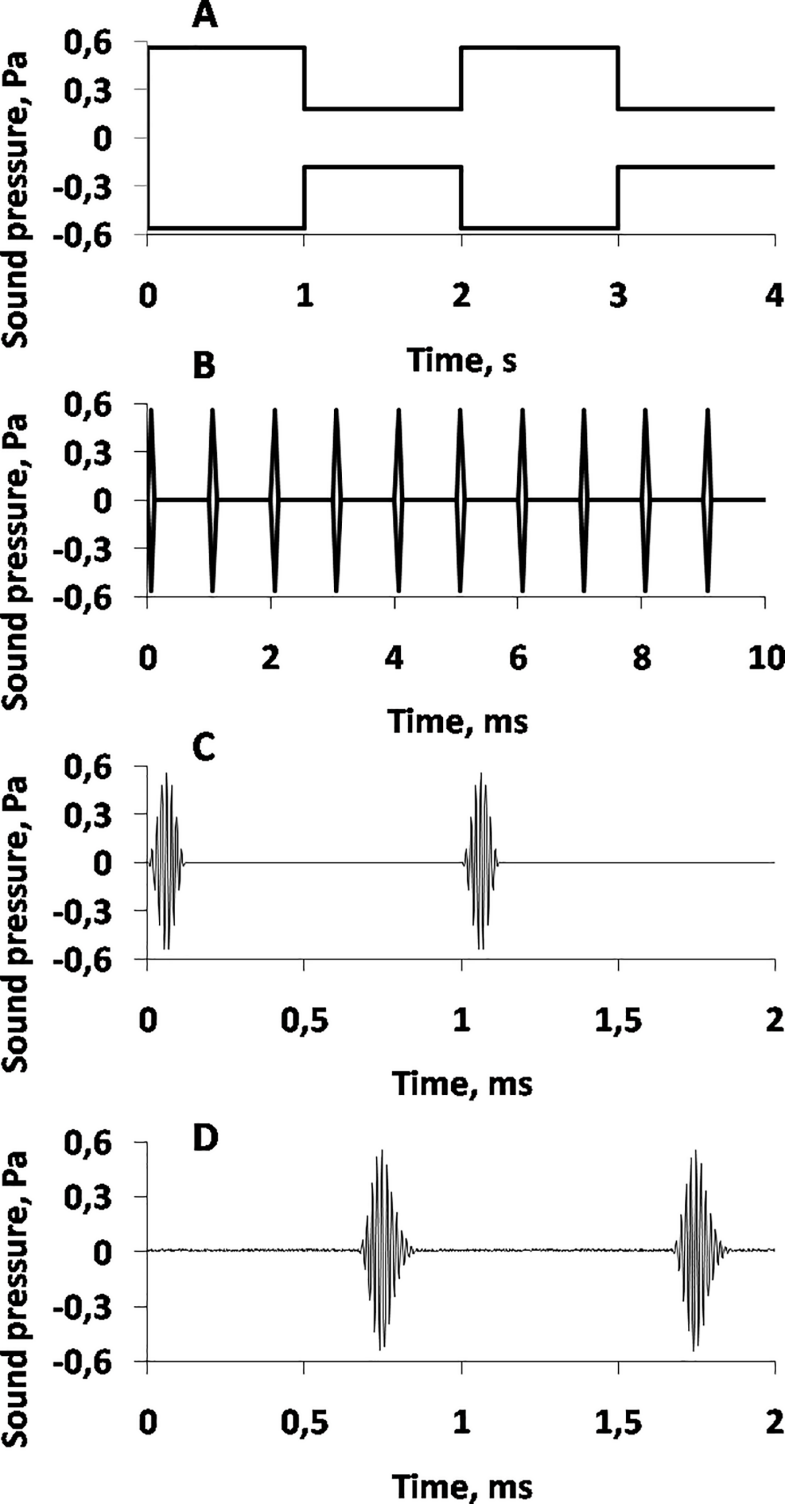
Stimulus waveforms at various time scales. A. Stimulus envelope at a compressed time scale. Stimulus level changes up/down every 1 s. B. Tone pip envelope. C. Tone pip waveform at an extended time scale (electric signal). D. Tone pip waveform at an extended time scale (acoustic signal).

The test stimuli were trains of short tone pips that periodically changed their level (Fig 1). The pips’ carrier frequency was 64 kHz with each pip enveloped by one cosine 125-μs cycle; thus, each pip contained 8 whole cycles of the carrier. The pip rate was 1000/s. The pips were presented continuously. The level of the tone pips was changed every 1s between two values with step sizes between 0 to 40 dB. Thus, during a trial, two stimulus levels were presented in interleaving manner. Tested stimulus levels spanned a range from 70–140 dB re 1 μPa. Each trial contained 500 cycles of up-down level changes and therefore lasted 1000 s.

All stimuli were digitally generated online at a sampling rate of 512 kHz by a standard personal computer using a custom-made program (Virtual Instrument) designed using LabVIEW software (National Instruments, Austin, TX). The synthesized signals were digital-to-analog converted by a NI DAQ-6251 acquisition board (National Instruments). To amplify and attenuate the test signal, a custom-made, low noise amplifier-attenuator with a 200-kHz passband and 50-Ohm output impedance was used. Sounds were played through a B&K 8104 transducer (Bruel&Kjaer, Naerum, Denmark).

The playback channel was calibrated by a receiving hydrophone (B&K 8103, Bruel&Kjaer) with a custom-made amplifier of 40-dB gain and a 200-kHz passband that was based on an AD820 chip (Analog Devices, Norwood, TX). The stimulus level was characterized in dB re 1 μPa RMS.

### Evoked potential recording

Brain potentials were picked up through surface F-E5G 10-mm gold-plated disk electrodes (Grass Technologies, Warwick, RI). The active electrode was positioned at the vertex 7 cm behind the blowhole and above the water surface. The reference electrode was positioned at the back.

Brain potentials were fed through shielded cables to an LP511 brain potential amplifier (Grass Technologies) with an 80-dB gain and a frequency passband of 100 to 3000 Hz. The potentials were digitized at a sampling rate of 16 kHz with a 16-bit analog-to-digital converter, which was one of the A/D channels of the NI DAQ-6251 acquisition board. The digitized signals were processed using a custom-made program (Virtual Instrument) designed using LabVIEW software (National Instruments).

### Data processing

The digitized brain potentials were averaged online, coherently with the changes of the signal level. Five hundred epochs were averaged to obtain one averaged brain response record. Further data processing was performed offline.

The averaged records were filtered by a 1-kHz tuned digital filter that was a Gabor unit of 1.75 ms equivalent rectangular duration (ERD) and 0.57 kHz equivalent rectangular bandwidth (ERB). The envelope of the filter output displayed the dynamics of RFR amplitude after upward or downward shifts of the stimulus level. With this data processing and the sampling rate of 16 kHz, estimates of response amplitude were available every 1/16 ms.

Each experiment with a certain combination of upward/downward shift of the stimulus level was repeated three times. The results of three identical experiments presented as response amplitude temporal dynamics were averaged with evaluation of both means and standard deviations (SD). Further processing was performed with these averaged data.

The amplitude-vs-time dependencies were approximated by exponent functions. To search for an approximating function, the time scale of the records was modified. In the modified scale, the samples were time-proportionally spaced by 1%. With this re-sampling, there were 232 samples in every log10 time unit. For each sample of the modified time scale, the response amplitude for a nearest sample on the original time scale was attributed. This procedure provided equal weights to all parts of the time scale when approximating the data by an exponent function.

The approximation function was defined as

$$A(t)={(A}_{0}-P)exp(-t/\tau )+P,$$ (1)

where *A*(*t*) is the exponent value at a time *t* after the stimulus level change, *A*_0_ is the exponent value at the instant of the stimulus level change, *τ* is the exponent time constant, and *P* is the plateau that is asymptotically approached at *t* →∞.

## Results

### Response waveforms and envelopes

**Fig 2:**
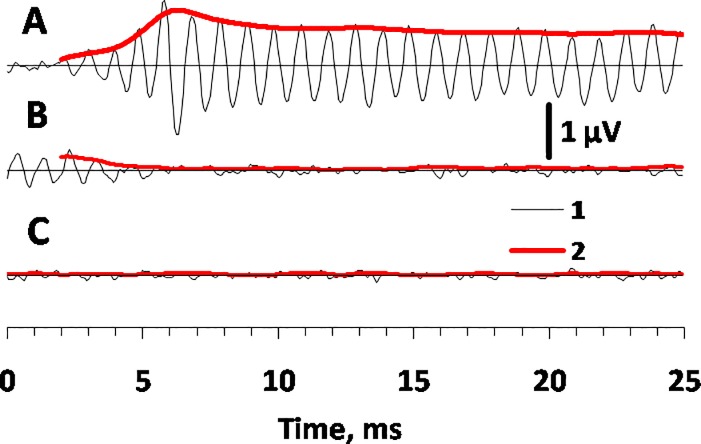
RFR waveforms and Gabor-filtered envelopes during the 25 ms after stimulus level changes. A. After a stimulus level increase from 100 to 120 dB re 1 μPa. B. After a stimulus level change from 120 to 100 dB re 1 μPa. 1 –waveform, 2 –envelope.

Response waveforms to the stimuli described above are exemplified in Fig 2. In order to extend the time scale and make the waveforms visible, windows as short as 25 ms after the stimulus level increase (A) and a 25 ms window after the stimulus level decrease (B) are presented. For comparison, a baseline record in the absence of a stimulus (C). This background level has amplitude of less than 0.05 μV. Below, the level of 0.05 μV was taken as an arbitrary response-present criterion; i.e., potential fluctuations less than 0.05 μV were considered as response-absent background noise of the record.

The stimuli produced a definite RFR as a succession of potential waves at a frequency of 1 kHz. The stimulus level increase after a previous lower-level stimulus resulted in a quick increase of the response amplitude (Fig 2A). The stimulus level decrease after the previous higher-level stimulus resulted in a quick decrease of the response amplitude. After 5.7 ms, the RFR amplitude was below the criterion specified above, so the response was considered completely suppressed (Fig 2B). The increase and decrease of responses featured a delay relative to the stimulus level changes: after a stimulus level increase, the response increase latency was approximately 2 ms (Fig 2A); after a stimulus level decrease, the response decrease delay was approximately 4 ms (Fig 2B). This 2 ms difference approximately corresponded to the duration of the main components of auditory brain responses (ABR) that composed the rhythmic RFR [17].

### Response amplitude dynamics

**Fig 3:**
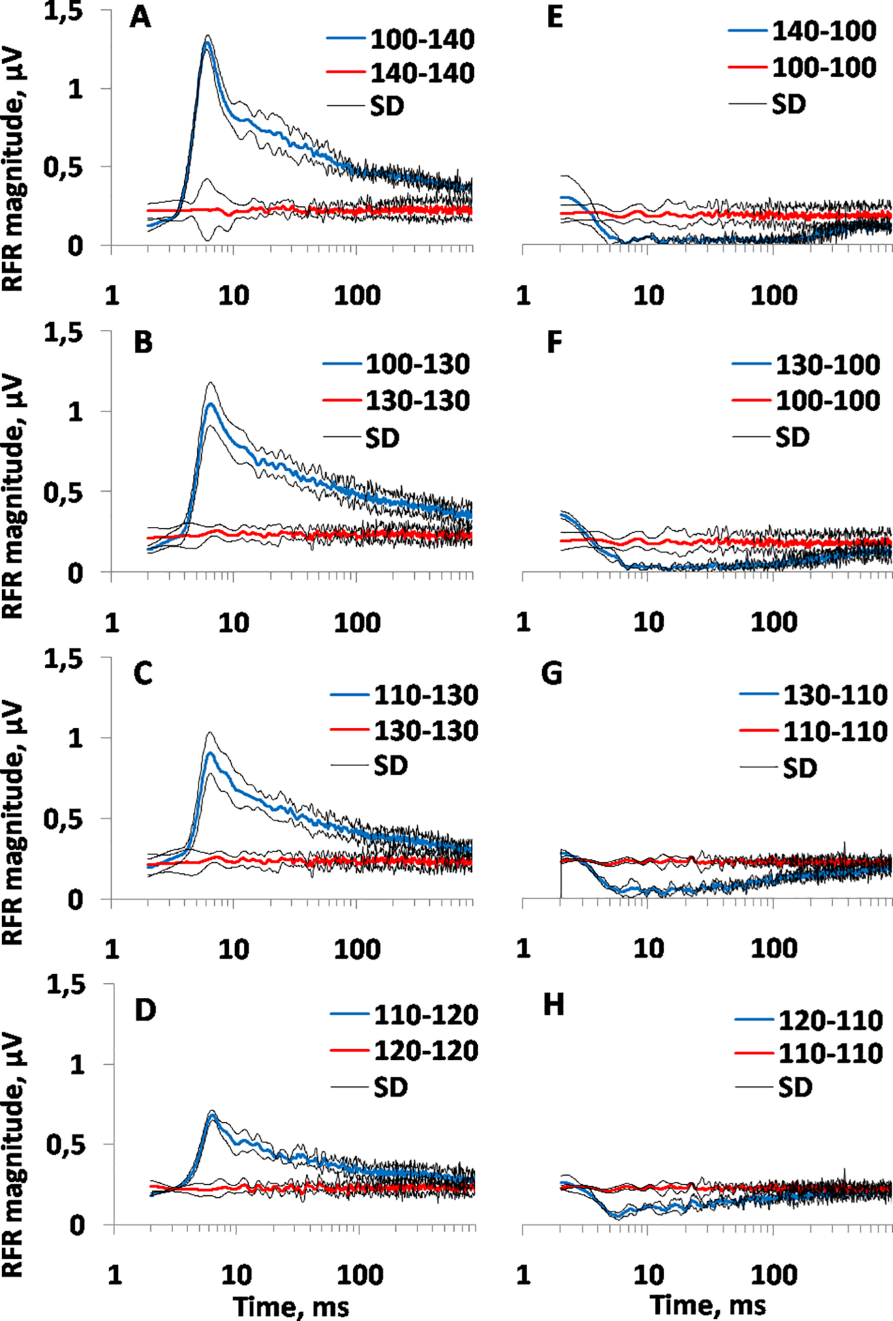
RFR magnitude as a function of time after stimulus level changes (“transient”) and steady state response magnitude to the same stimulus level. Each record presents the mean and SD of a Gabor-filtered envelope (see Fig 2) of averaged data from three experiments. The left column–records after level changes from 40-dB (A) to 10-dB (D) increase; the right panel–records from 40-dB (E) to 10-dB (H) decrease. The legends show the levels before and during the recording window, e.g., “100–140”–level change from 100 (before the recording window) to 140 dB (during the recording window) re 1 μPa, “140–140”–steady-state level of 140 dB re 1 μPa. Thin black lines (SD)–ranges of standard deviations.

Response dynamics at up-and-down variation of the stimulus level are presented in Fig 3 at several “higher” and “lower” stimulus levels. For comparison, response dynamics to steady-state stimulus level (a constant level throughout the data-collection time, both before and during the recording window) are presented.

After a transition from a lower to higher stimulus level (increase), the response amplitude increased quickly and then decreased slowly. During the 1000 ms increased stimulus, the response amplitude decreased by 3 to 4 times as compared to the amplitude immediately after the stimulus increase and almost reached the amplitude of the response to the steady-state (i.e., continuous with no transient) stimulus of the same level (Fig 3A).

After a transition from a higher to lower stimulus level (decrease), the response amplitude fell quickly and later recovered slowly (Fig 3B). When the span between the higher and lower levels was 20 dB or more, immediately after the signal level decrease, the response was considered completely suppressed according to the criterion specified above (see Fig 2); then the response recovered. The duration of the complete response suppression varied from approximately 20 ms after a stimulus decrease by 20 dB to approximately 200 ms after a stimulus decrease by 40 dB. After a decrease by 10 dB, the response was suppressed partially. In all cases, during the 1000 ms stimulus, the response amplitude almost reached that of the steady-state stimulus of the same level.

**Fig 4:**
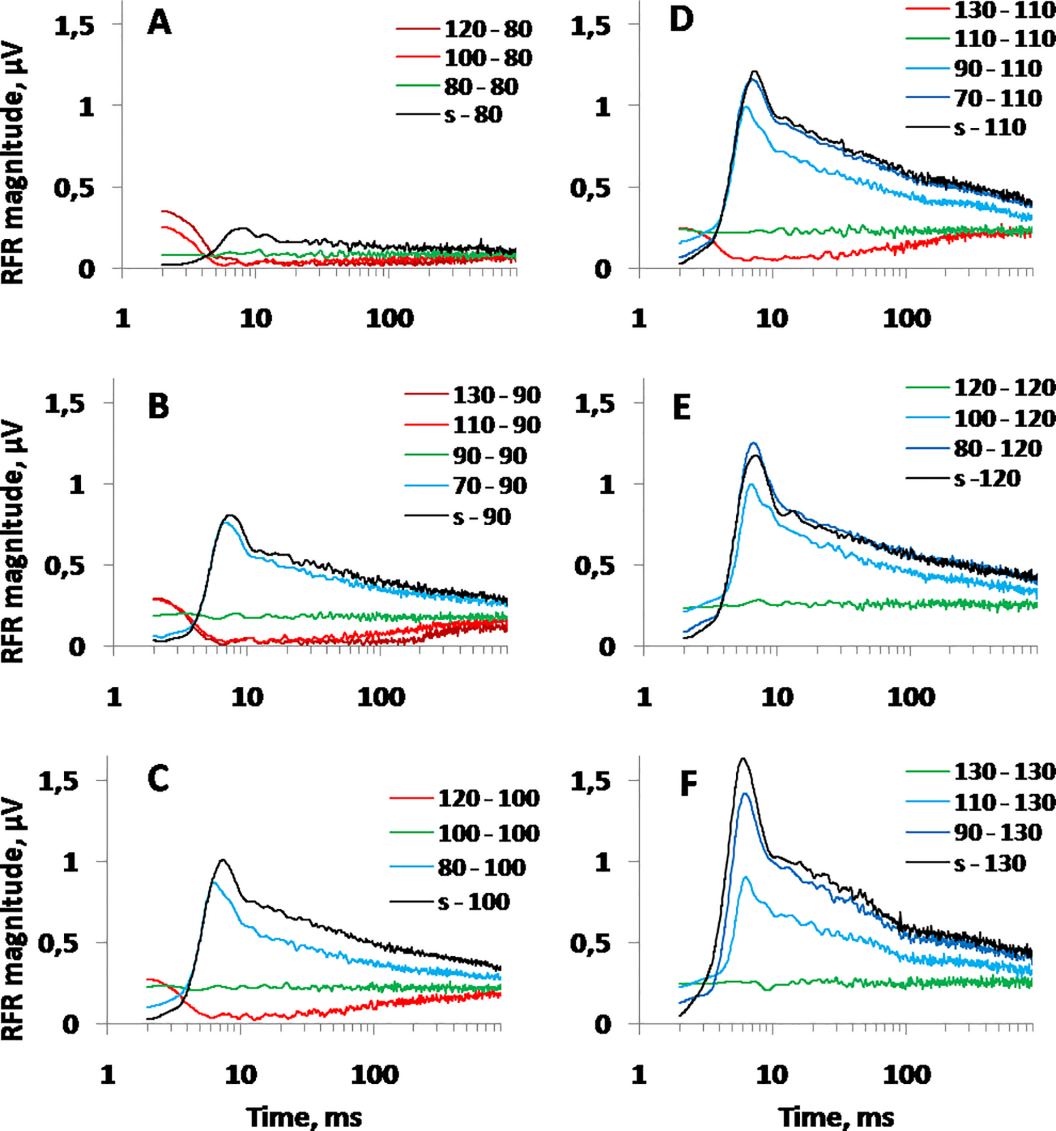
**RFR magnitude as a function of time after stimulus level changes, keeping level change span as a parameter, for test signal levels from 80 (A) to 130 dB SPL (F).** In each panel, the previous and current stimulus levels are indicated for each of the plots; *s*–silence; equal previous and current levels mean steady-state stimulation. For example, “120–80”–stimulus 80 dB re 1 μPa after 120 dB re 1 μPa; “80–80”–steady-state stimulus of 80 dB re 1 μPa; “s-80”–stimulus 80 dB after silent (no-stimulus).

In detail, the dynamics of the response amplitude during a 1000 ms presentation of different stimulus levels was investigated for stimulus levels from 70 to 130 dB re 1 μPa varied with 10-dB steps. These “test” stimuli (i.e., stimuli that produced the recorded responses) were alternated with 1000-ms-long stimulus levels varied with 10-dB steps from –40 to +40 dB relative to the “test” level but not higher than 130 dB SPL and not lower than 70 dB SPL. The alternating periods could also be silent (no stimulus). Notably, among the combinations of the test and alternative stimulus levels, there were combinations in which these two levels were equal. This combination produced the steady-state stimulation when a certain stimulus level was kept constant throughout the entire1000 s trial. Each combination of the “test” and alternative stimulus levels was repeated three times. The results are presented in Fig 4, which displays the response amplitudes to the “test” level as a function of time after a signal level change up or down.

The functions presented in Fig 3 and Fig 4 demonstrated the following:

- After a stimulus level increase, the response amplitude increased quickly with a subsequent slow decay. The more the level increased, the higher the response amplitude increased.
- After a stimulus level decrease, the response amplitude was suppressed with a subsequent slow recovery. The more the level decreased, the deeper the response suppression was.
- When there was no level change (the steady-state stimulation), the response amplitude depended on stimulus level (the higher the level, the higher the response amplitude, from approximately 0.08 μV at 80 dB to 0.25 μV at 130 dB SPL) but was approximately constant during the 1000 ms analysis window.
- At the end of the 1000 ms window, the response amplitude approached, but did not reach, the amplitude characteristic of the steady-state stimulation.

To quantitatively characterize the temporal dynamics of the RFR after the stimulus increase/decrease, the dependence of the RFR amplitude on stimulation time was approximated by an exponential function within a time range from 10 to 1000 ms. We have previously shown in the beluga that an RFR decay during prolonged stimulation may be approximated by two exponents with time constants of 30 to 80 ms and 3.1 to 17.6 s [16]. The time constant of several seconds is beyond the duration of the 1000 ms analysis window in the present study. Therefore, we used approximations with one exponent for 1000 ms records.

**Fig 5:**
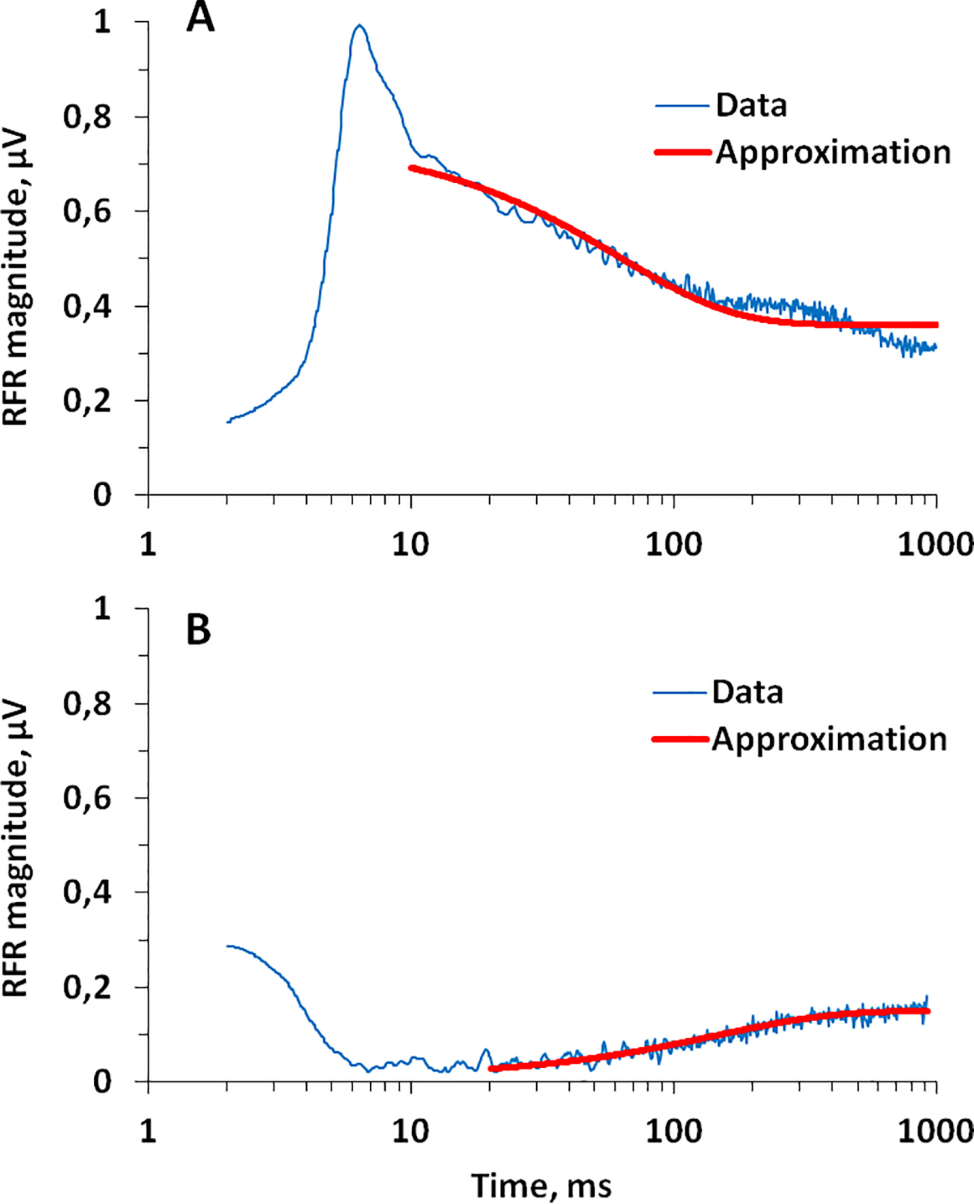
Examples of approximations of RFR magnitude dependence on time by exponents. A. Stimulus 110 dB after 90 dB re 1 μPa; approximation of a segment 10–1000 ms after a stimulus level change by an exponent of time constant of 62 ms. B. Stimulus 90 dB after 110 dB re 1 μPa; approximation of a segment 20–1000 ms after a stimulus level change by an exponent of time constant of 146 ms.

For stimulus level increases, the approximation was performed within a time range from 10 to 1000 ms and ignoring the transient on-response within 10 ms after the level increase. For stimulus level decreases, the approximation was performed ignoring the time of complete response suppression; the complete suppression was determined according to the criterion specified above (see Results: Response waveforms and envelopes). With this constrain, approximation was performed within time ranges of 7 to 1000 ms, 20 to 1000 ms, 70 to 1000 ms, or 200 to 1000 ms for signal decrease spans of 10, 20, 30, or 40 dB, respectively. The parameters were iteratively adjusted to obtain the best fit of the equation to the experimental data according to the least-mean-square criterion. Examples of such approximation for a signal level increase from 90 to 110 dB, and a decrease from 110 to 90 dB are presented in Fig 5A and 5B, respectively. In these particular examples, the approximation resulted in time constants of 62 ms for the response decay after the stimulus level increase (Fig 5A), and 146 ms for the response recovery after the stimulus level decrease (Fig 5B). Approximations and time-constant evaluations were performed for the averaged amplitude-vs-time functions, i.e., one result for each stimulus combination, so no data scatter estimates (standard deviations) were available at this stage of data processing.

**Fig 6:**
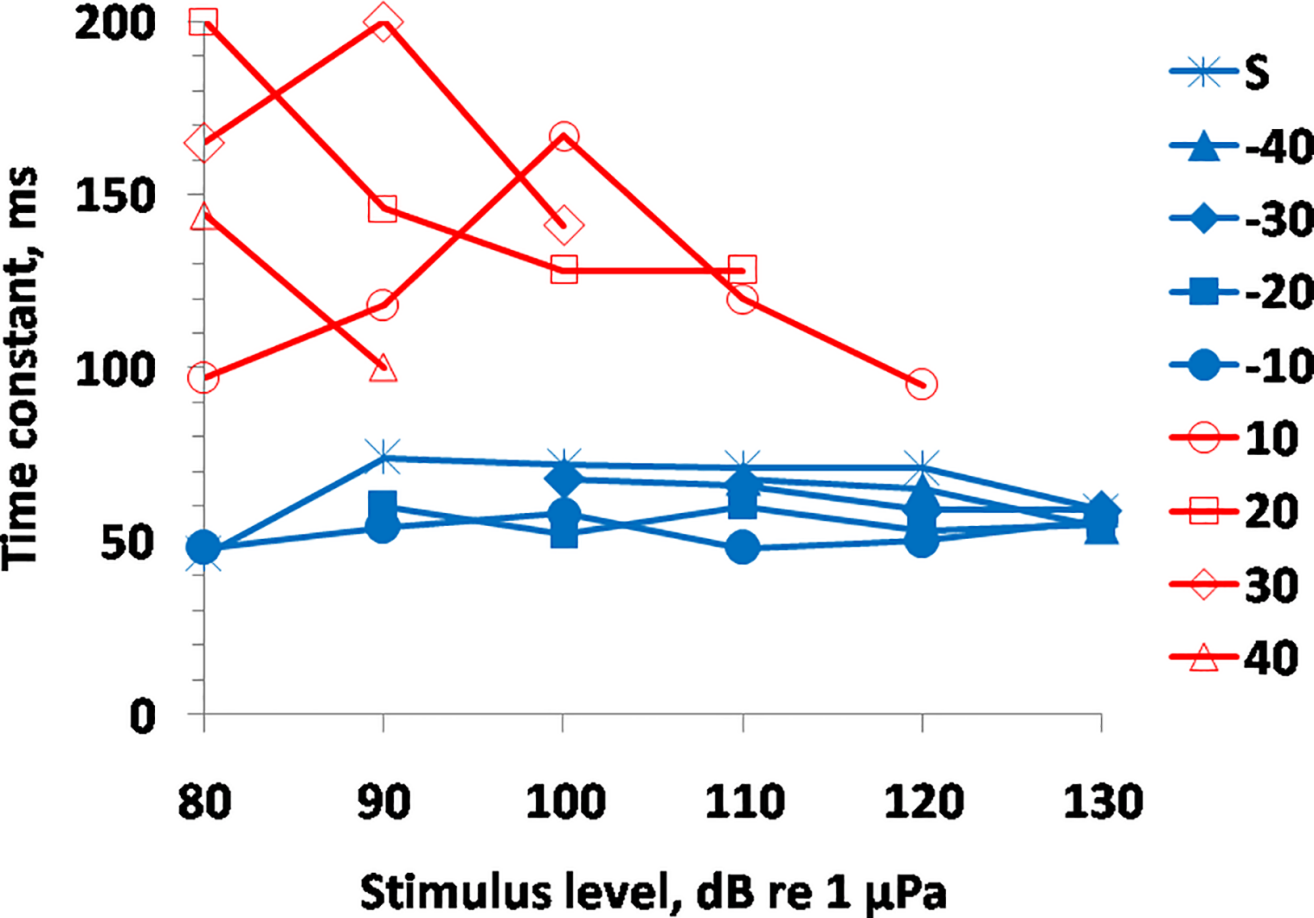
Time constants of approximating exponents as functions of stimulus level, keeping the relative level of the previous stimulus as a parameter; *s* (silence)–no previous stimulus. For example, “40”–previous stimulus level 40 dB above the current stimulus level; “-40”—previous stimulus level 40 dB below the current stimulus level.

The results of the approximations featured a substantial scatter of time constant estimates (Fig 6). Nevertheless, there was a separation of the obtained *τ* values into two main groups. After a stimulus level increase (including stimulus onset after silence), the *τ* values were below 75 ms; after a stimulus level decrease, the *τ* values were above 95 ms. Within each of these groups, the *τ* values featured no systematic dependence on either stimulus level or increase/decrease span. The means and standard errors for the increase and decrease groups were 59.4 ± 1.8 and 139.2 ± 9.9 ms, respectively, and the means differed significantly by *t*-test (*t = –*2.65; *p* < 0.001). Thus, after a stimulus level decrease, the recovery of the response amplitude was more than twice as slow as the decay after a stimulus level increase.

### Response dependence on stimulus level and increase/decrease span

**Fig 7:**
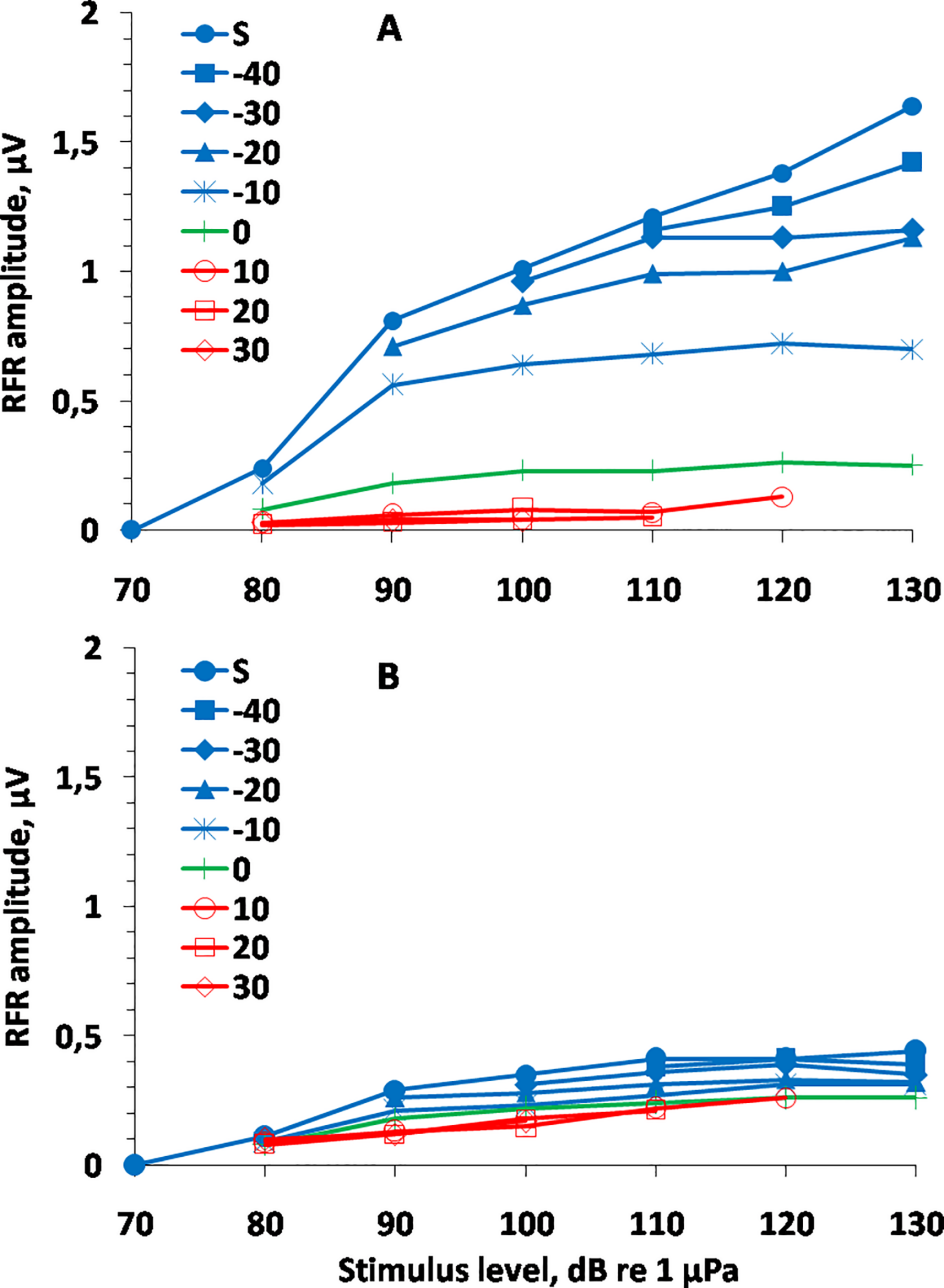
RFR magnitudes as functions of the stimulus level, keeping the relative level of the previous stimulus as a parameter; *s* (silence)–no previous stimulus. For example, “–40”–previous stimulus level 40 dB below the current stimulus level; “30”—previous stimulus level 30 dB above the current stimulus level. A. within a time window 6.5 to 7 ms after stimulus level change. B. within a time window 900 to 1000 ms after stimulus level change.

After up/down changes of the stimulus level, the response amplitude decayed after an initial rise (if the signal level increased) and recovered after an initial fall (if the signal level decreased). As a result, at the end of the 1000 ms analysis window, the response amplitudes to the higher and lower stimulus levels differed much less than those immediately after the stimulus level change. Fig 7 presents the response amplitude dependence on both the stimulus level and span of level change. The dependencies are presented both for amplitudes 6.5 to 7 ms after the level change (Fig 7A) and for the end of the analyzed window 900^th^ to 1000^th^ ms after the level change (Fig 7B). Shortly after the level change, the response amplitudes substantially depended both on the current stimulus level (as manifested by slopes of the plots) and on the span of the level changes (as manifested by different plot positions) (Fig 7A). The responses at the end of the analysis window (Fig 7B) were little dependent on the stimulus level above 90 dB re 1 μPa (as manifested by nearly flat plots) or on the span of the level changes (as manifested by all the plots grouping close to one another).

Thus, during the 1000 ms presentation of stimuli of a given level, the response amplitude adapted toward a narrow range of signal levels. In the described experiments, this range was from 0.12 μV (a stimulus level of 90 dB after a 30 dB level decrease) to 0.44 μV (a stimulus level of`130 dB after a stimulus onset in silence). However, the response amplitude still did not reach an asymptotic value during the 1000 ms analysis window. During steady-state stimulation, the dependence of the response amplitude on the stimulus level was even less: from 0.18 μV at a level of 90 dB to 0.26 μV at a level of 130 dB re 1 μPa.

For comparison, shortly after the level change within the same range (from 90 to 130 dB re 1 μPa), the responses varied considerably more: from 0.04 μV (a stimulus level of 90 dB after a 30 dB level decrease) to 1.64 μV (a stimulus level of 130 dB after onset in silence).

## Discussion

### Role of adaptation in the effects of varying stimulus level

The data presented above demonstrate that during prolonged auditory stimulation, auditory responses vary depending on several stimulation conditions: current stimulus level, previous stimulus level, span between the previous and current stimulus levels, and time after stimulus level change. Shortly after a level change, the response amplitude substantially depended both on the current stimulus level and on the direction and span of the level change. Later after the level change, these dependencies diminished. Thus, during the presentation of a given stimulus level, the responses featured a sort of amplitude equalization. This equalization could be attributed to adaptation processes.

Notably, the “equalized” response amplitude was much less than shortly after a stimulus onset or increase. However, any stimulus level change resulted in a substantial increase or decrease of the response amplitude. Thus, while little dependent on the current stimulus level, the responses were substantially dependent on stimulus level changes. This fact is in agreement with the idea that the differential sensitivity of the auditory system is maintained against the background of the adapting signals [2]. This response property may reflect an adjustment of the dynamic range of the auditory system to the current SPL of the auditory environment.

### Time course of the response variation during stimulation

Our previous investigation [16] showed that in the beluga, the time course of the response amplitude during prolonged stimulation can be satisfactorily approximated by a combination of two exponents: a “short” exponent with a time constant from 30 to 80 ms and a “long” exponent with a time constant from 3.1 to 17.6 s. These two exponents may reflect two adaptation processes known as the short-term and long-term adaptation. In the present study, we ignored the “long” adaptation because response variations were investigated within a time window of no longer than 1000 ms. Within this 1000 ms window, the adaptation process was successfully approximated by one exponent. This approximation allowed for comparison of adaptation time courses during the 1000 ms analysis window at different stimulation conditions.

Investigation of adaptation when a stimulus periodically switched from lower to higher level and back has been performed in auditory-nerve fibers [11, 18]. It has been shown that the time courses of these two opposite processes are different: the time constants of adaptation after upward switches were about one-half the time constant for downward switches (on average, 131 and 272 ms, respectively) [18]. In the present study for AEP in the whale, time courses were almost twice shorter (59 and 139 ms, respectively) but with the same ratio of time constants for upward and downward changes of the signal level: a response recovery after a stimulus level decrease proceeded more than twice as slow as response decay after a stimulus level increase.

Short time constants of adaptation in the whale may be one of aspects of high temporal resolution of hearing in odontocetes [13–15]. Different time constants for adaptation after upward and downward switch may indicate either the action of different mechanisms of adaptation for increased and decreased stimulus levels, or non-linearity of the adaptation mechanism, which may function at a different speed depending on direction of the process. The first suggestion (two different mechanisms) seems less probable: if a mechanism functioned either only after sound increase or only after decrease, it might be saturated quickly. Nonlinearity of a common adaptation mechanism seems a more realistic explanation.

Considering unique properties of the cetacean’s hearing, comparison of the temporal adaptation dynamics in the beluga with that in other mammals might be of special interest. However, investigations of adaptation have been performed in a limited number of mammalian species, and the methods in these experiments were different. It makes difficult to compare the results obtained in this study with those obtained other studies. However, it may be noted that the time course of adaptation constants measured in the beluga whale (time constants of 59.4 and 139.2 ms for upward and downward signal level changes, respectively) is closer to the so-called short-term adaptation (time-constants of tens of ms) rather than to rapid adaptation (time constants of several ms) or long-term adaptation (time constants of seconds or tens of seconds) found in other mammals [1, 2, 4, 6, 19–21]. The physiological nature of the short-term adaptation is still debatable. However, a majority of models ascribe adaptation to depletion and subsequent restoration of transmitter in several synaptic levels of the auditory system [22–24].

Notably, both after a sound level increase and decrease, the responses almost stabilized during an analysis time as short as 1 s. Based on this property, the auditory system of the beluga (and, hypothetically, other odontocetes) may be characterized as quickly flexible and capable of quick adjustment to the current auditory scene.

## Supporting information

S1 Table Readaptation processes at signal levels 90 to 110 dB SPL. For each signal level, three repetitions (Block 1 to Block 3) and mean are presented.
(XLSX)

S2 Table Readaptation processes at signal levels 120 to 140 dB SPL. For each signal level, three repetitions (Block 1 to Block 3) and mean are presented.
(XLSX)
